# Quantitative study on hepatic genotoxicity of neodymium and its molecular mechanisms based on Benchmark Dose method

**DOI:** 10.3389/fphar.2024.1484111

**Published:** 2024-12-17

**Authors:** Ning Wang

**Affiliations:** Institute of Chemical Toxicity Testing/NHC Specialty Laboratory of Food, Safety Risk Assessment and Standard Development/State Environmental Protection Key Laboratory of Environmental Health Impact Assessment of Emerging Contaminants, Shanghai Municipal Center for Disease Control and Prevention, Shanghai, China

**Keywords:** neodymium nitrate, alkaline comet assay, P53 signaling pathway, genotoxic biomarkers, Benchmark Dose method

## Abstract

**Introduction:**

Neodymium, a rare earth element, has been shown to induce genotoxicity in mice, but the molecular mechanisms behind this effect are not fully understood. This study aims to investigate the genotoxic effects of intragastric administration of neodymium nitrate (Nd(NO_3_)_3_) over 28 consecutive days and to elucidate the underlying molecular mechanisms.

**Methods:**

We detected the content of neodymium in mouse liver tissue using ICP-MS and assessed the percentage of tail DNA in mouse hepatocytes using the alkaline comet assay to evaluate genotoxicity. Additionally, we evaluated genetic toxicological biomarkers (reactive oxygen species (ROS), 8-hydroxy-2′-deoxyguanosine (8-OHdG), and γ-H2AX) and the expression levels of genes related to the p53 pathway in the mouse liver.

**Results:**

Our findings indicate a potential accumulation of (Nd(NO_3_)_3_) in the livers of mice, leading to DNA double-strand breaks in hepatocytes, as evidenced by comet and γ-H2AX assays. Nd(NO3)3 significantly increased the percentage of tail DNA in hepatocytes and upregulated the expression of molecules related to the p53 pathway, including ATM, Wip1, ATR, Chk2, MDM2, p53, p21, and NF-κB, at the transcriptional level. The treatment also effectively triggered the production of ROS, 8-OHdG, and γ-H2AX in liver tissue.

**Discussion:**

These results suggest that (Nd(NO_3_)_3_) induces hepatic genotoxicity and injury in mice and modulates the expression of genes associated with DNA damage response, carcinogenesis, and inflammatory processes. The study provides insights into the molecular mechanisms by which neodymium nitrate exerts its genotoxic effects and underscores the importance of further investigating the potential health risks associated with neodymium exposure.

## 1 Introduction

Rare earth elements (REEs) encompass the 17 elements of the lanthanide series, including lanthanum, cerium, praseodymium, neodymium (Nd), and others, with scandium and yttrium often included due to their similar chemical properties. Their unique physical and chemical attributes, such as high reactivity and low ignition points, have led to broad applications across various sectors, including industry, agriculture, medicine, and high-tech fields (Agency, 2012). Notably, REEs are integral to the production of glass, ceramics, magnets, electronics, superconductors, and lasers, with neodymium’s role in manufacturing magnets for speakers, computer hard drives, wind turbines, and hybrid vehicles being particularly significant (Rim et al., 2013). Additionally, fertilizers enriched with lanthanum, cerium, and neodymium have been reported to boost crop yields in China (EFSA Scientific Committee, 2011; Hu et al., 2004).

However, the surge in REE mining and industrial use has resulted in their widespread release into the environment, raising concerns about potential accumulation and pollution in soil, vegetation, water, and air (EFSA Scientific Committee, 2011; Hu et al., 2004; Cao et al., 2014; H. M, 2019). Neodymium, detected as the third most abundant REE in Chinese soil with a background value of 25.1 mg/kg, follows cerium at 64.7 mg/kg and lanthanum at 37.4 mg/kg, and is considered potentially highly toxic (Berg et al., 2011). This has heightened the interest in neodymium’s potential benefits and the associated environmental and health risks (Xiaofei, 2013; Freitas et al., 2020).

Human exposure to REEs occurs through the digestive tract, respiratory system, and skin, with detectable levels in blood, urine, and hair (Zhao et al., 2011). Evidence from animal studies and occupational exposure suggests that REEs can bioaccumulate and cause damage to vital organs such as the lungs, liver, and brain (Liu, 2008). Specifically, neodymium has been implicated in oxidative damage within hepatocytes, affecting both the nuclei and mitochondria (Huang et al., 2011). The liver, lungs, and blood are recognized as primary targets for REE-induced toxicity (Sturla Lompré et al., 2021), with light REEs, including neodymium, tending to accumulate in the liver and spleen (Ruan, 2011). Studies have also linked Nd(NO_3_)_3_ exposure to liver tissue damage, oxidative stress, and inflammation in rats (Liao, 2009; Gao, 2020; Fei et al., 2011).

Despite the evidence of neodymium’s genotoxic potential, the underlying mechanisms and long-term health impacts remain unclear, underscoring the need for further research.

This study posits that neodymium nitrate’s genotoxicity may be mediated through the p53 signaling pathway, a canonical route in DNA damage response. Using the OECD-recommended *in vivo* comet assay, we assessed neodymium nitrate’s genotoxicity and investigated the correlation between hepatic neodymium content and subacute genotoxic effects. We further explored the impact of neodymium nitrate on oxidative stress markers (ROS, 8-OHdG, γ-H2AX) and p53 pathway components to delineate the mechanism of its genotoxicity (The assessment of mutagenicity health protection branch mutagenicity guidelines, 1993; Hirano and Suzuki, 1996).

The determination of a Point of Departure (PoD) for human health risk assessment is a critical aspect of quantitative genetic evaluation. The Benchmark Dose (BMD) approach, endorsed by the International Workshop on Genotoxicity Testing (IWGT), is a prominent method for deriving PoD thresholds (MacGregor, 2015; Pottenger and Gollapudi, 2010). The BMD is calculated using specific software and models to identify the dose at which a predetermined response level change, the benchmark response (BMR), occurs (Hardy et al., 2017; Chain and Knutsen, 2018; Izadi, 2012).

The p53 gene, a pivotal tumor suppressor and the “guardian of the genome,” is central to cellular responses to DNA damage and other stressors (Chen, 2019; Lahav et al., 2004). This study examines the role of p53 pathway components in neodymium nitrate-induced DNA damage and p53 response modulation. Adhering to OECD guidelines (OECD, 2016), we evaluated hepatocyte DNA damage and oxidative stress markers to assess neodymium nitrate’s genotoxic potential. Our research aims to innovate toxicity assessment and risk prediction methodologies, enhancing chemical risk analysis, disease surveillance, and pharmaceutical research.

## 2 Materials and methods

### 2.1 Chemicals and reagents

Neodymium nitrate hexahydrate [Nd(NO_3_)_3_·6H_2_O] was obtained from Sigma-Aldrich with a CAS number of 16,454–60-7 and a purity of 99.9%. Ethyl methanesulfonate, used as a positive control, was also sourced from a reliable chemical supplier.

### 2.2 Animal model and treatment regimen

Six-week-old ICR mice, weighing 29egimenourced from a reliable chemical Weitong Lihua Experimental Animal Technology Co., Ltd. (China). Thirty male mice were housed under controlled conditions with a temperature of 22°C ± 2°C, relative humidity of 60% ± 5%, and a 12-h light/dark cycle. They were provided with food and water *ad libitum* and allowed a 5-day acclimation period prior to the experiment. The study was approved by the International Ethics Committee on Animal Welfare, and all procedures were conducted in accordance with their guidelines.

The mice were randomly assigned into seven groups: a negative control, a positive control, and five experimental groups receiving Nd(NO_3_)_3_ at dosages of 7, 27, and 55 mg/kg body weight (n = 6 per group). The dosages for the 28-day subacute toxicity study were determined based on an acute genotoxicity test using a dose of 39 mg/kg for male mice. Nd(NO_3_)_3_ was administered daily by gavage for 28 days. The negative control group received an equivalent volume of purified water, while the positive control group received ethyl methanesulfonate (200 mg/kg) in purified water. Daily observations of symptoms and mortality were recorded throughout the study.

At the conclusion of the treatment period, blood was collected via the retro-orbital plexus method for neodymium content analysis. Mice were euthanized by cervical dislocation, and liver samples were collected for comet assay, qRT-PCR, ELISA, and histopathological examination.

### 2.3 *In Vivo* comet assay

The *in vivo* comet assay was performed in accordance with the OECD Test Guideline 489 (OECD, 2016). Three hours following the final administration, mice were euthanized, and single liver cells were isolated from the left lateral lobe. The procedure utilized a commercial comet assay kit (Catalog Number: BR-0904, BIOLAB Company). Low-melting agarose was prepared at a concentration of 0.5% (w/v) in D-PBS (lacking Ca^2+^, Mg^2+^, and phenol red) and heated using a microwave oven. This agarose was maintained at 37°C–45°C and was discarded after a single use. Standard agarose gels were prepared by heating routine melting point agarose in phosphate-buffered saline (pH 7.0 to 7.4, without Ca^2+^, Mg^2+^, and phenol red) to achieve concentrations between 1.0% and 1.5% (w/v).

The lysis solution was prepared using pure water, with a final concentration of 100 mM EDTA-2Na, 10 mM tris-base, and 2.5 M NaCl. The pH of this solution was adjusted to 10 with 5 M NaOH or 6 M HCl and was stored at 4°C–8°C. Prior to the experiment, 1% (v/v) triton-X100% and 10% (v/v) DMSO were added to the lysis solution, which was then refrigerated at 4°C–8°C for a minimum of 30 min. The alkaline unwinding solution and electrophoresis solution were also prepared with pure water, reaching final concentrations of 1 mM EDTA-2Na and 300 mM NaOH, with a pH of at least 13. These solutions were stored at 4°C–8°C, and their pH was verified before use.

The neutralization solution was prepared with pure water, achieving a final concentration of 0.4 M tris-base (pH adjusted to 7.5 with 5 M NaOH or 6 M HCl), and was kept at 4°C–8°C. EDTA-2Na was dissolved in HBSS (without Ca^2+^, Mg^2+^, and phenol red) to a final concentration of 20 mM EDTA-2Na, with the pH adjusted to 7.5 using 5 M NaOH or 6 M HCl. This solution was stored at 4°C–8°C, and 10% DMSO was added immediately before use. DNA fluorescent dye (Gelred) was prepared and applied according to the manufacturer’s instructions.

Liver cells were embedded in low-melting agarose and lysed at 4°C for 1 h. Post-lysis, slides were neutralized, incubated in an alkaline solution to facilitate DNA unwinding, and then subjected to electrophoresis. The slides were subsequently stained with Gelred, rinsed, and analyzed using a Leica DM3000 fluorescence microscope equipped with a fiber optic lamp and a ×200 magnification lens. All slides, including negative and positive controls, were evaluated, with a minimum of 150 cells examined per sample (each tissue from each animal). At least five animals per dose were utilized, with 150 cells counted per animal. Additional fields of view were observed at the same density to ensure that comet tails did not overlap, and the edges of the slides were not considered in the scoring. The occurrence of “hedgehog” cells was noted separately. The Comet Intelligence Analysis software was employed to assess DNA damage by quantifying the percentage of tail DNA in 150 cells per sample.

### 2.4 Determination of neodymium content in livers by ICP-MS

Liver tissue samples were digested in microwave digestion tubes with 5 mL of trace metal-grade nitric acid (CNW Technologies) using a microwave digestion system. The digestion program consisted of three steps: ① 5 min at 120°C with a 5 min hold; ② 5 min at 150°C with a 10 min hold; ③ 5 min at 190°C with a 20 min hold. After cooling, samples were subjected to acid removal at 120°C for approximately 30 min, then diluted to 25.0 mL with ultrapure water (18.2 MΩ cm, Milli-Q), and mixed thoroughly.

Analysis was performed using Inductively Coupled Plasma Mass Spectrometry (ICP-MS; Agilent 8900 ICP-MS/MS, Agilent Technologies, USA) with the following parameters: RF power 1500W, concentric nebulizer, plasma gas flow 15 L/min, nickel sampling cone/skimmer cone, carrier gas flow 0.80 L/min, sampling depth 10 mm, auxiliary gas flow 0.40 L/min, peak hopping acquisition mode, helium flow 4.3 mL/min, automatic detection mode, atomization chamber temperature 2°C, three measurement points per peak, sample lift rate 0.3 r/s, and three repetitions. Indium (50 ng/mL) served as the internal standard. The neodymium detection limit was 0.0018 ng/mL, with data expressed as nanograms per Gram of fresh tissue.

### 2.5 Liver γ-H2AX Content by ELISA

Approximately 50 mg of liver tissue was snap-frozen in liquid nitrogen for γ-H2AX content determination using an ELISA kit (Mouse γH2AX ELISA KIT, Shanghai Enzyme Union Biotechnology Co., LTD.). Liver samples were homogenized in Hank’s Balanced Salt Mixture (D-Hanks, without Ca^2+^ and Mg^2+^, without phenol red, pH 7.4) and centrifuged at 2000–3,000 rpm for 20 min. The supernatant was collected for immediate testing or stored at −20°C to avoid repeated freezing and thawing. Protein content was measured by the Lowry method (Kashyap et al., 1980), with triplicate analyses for each sample.

### 2.6 Liver ROS content by ELISA

ROS content in liver tissue was determined using a similar ELISA protocol (Mouse ROS ELISA Kit, Shanghai Enzyme-Linked Biotechnology Co., Ltd.). Samples were processed and analyzed as described for γ-H2AX, with the same conditions for homogenization, centrifugation, and storage.

### 2.7 Liver 8-OHdG content by ELISA

The content of 8-OHdG in liver tissue was measured by ELISA (Mouse 8-OHdG ELISA Kit, Shanghai Enzyme-Linked Biotechnology Co., Ltd.) following the same homogenization, centrifugation, and storage procedures as the previous assays. The protein content was determined by the Lowry method, and triplicate analyses were conducted for each sample.

### 2.8 cDNA sample preparation from mouse liver

Total RNA was extracted using the Total RNA Extraction Kit from TIANGEN BIOTECH (Catalog Number: DP451, Item Number: 4,992,562, Lot Number: Y1209). The purity of the RNA was confirmed with an A260/A280 ratio of 2.1, indicating that it was free from protein contamination. Reverse transcription was performed using the FastKing gDNA Dispelling RT SuperMix from TIANGEN BIOTECH (Catalog Number: KR118-02, Lot Number: X1010). The resulting cDNA was resuspended in Tris-HCl buffer (pH 7.2) and stored at 4°C. mRNA expression levels were determined by qRT-PCR using the SuperReal PreMix Plus (SYBR Green, Catalog Number: FP205-02, Lot Number: X1018) from TIANGEN BIOTECH, with β-actin serving as the reference gene for analysis. Primer sequences were identified through a literature search (Ruan, 2011) and synthesized by Sangon Biotech. Primer details are presented in Table 1.

**TABLE 1:** Primer Pairs Used for Gene Expression Analysis by qRT-PCR.

Gene name	Primer Sequence(5′to 3′)	Base pair count	Provide total quantity(OD)	Purification method
ATM gene	CCAAGATGGCAGTGAACCAGAC-F	24	2OD	HPLC
	ATGCTGGACAGCTATGGTGGAG-R	23	2OD	HPLC
Wip1 gene	TCACAGTGGACCTGTCAGAAGG-F	24	2OD	HPLC
	AGAGTGTGGACACTGGTGTCTG-R	23	2OD	HPLC
ATR gene	GAAAGAGGCTCCTACCAACGAG-F	23	2OD	HPLC
	CAACTGTCACCTGGAGACTTGC-R	23	2OD	HPLC
Chk2 gene	GAGGTTCTTGTCTCCAACGGGA-F	23	2OD	HPLC
	ATCCTTCAGGGACACTTGGGTC-R	23	2OD	HPLC
MDM2 gene	CCGAGTTTCTCTGTGAAGGAGC-F	24	2OD	HPLC
	GTCTGCTCTCACTCAGCGATGT-R	23	2OD	HPLC
β-actin gene	CATTGCTGACAGGATGCAGAAGG-F	24	2OD	HPLC
	TGCTGGAAGGTGGACAGTGAGG-R	23	2OD	HPLC
p53 gene	AGAGACCGCCGTACAGAAGA-F	22	2OD	HPLC
	GCATGGGCATCCTTTAACTC-R	21	2OD	HPLC
p21 gene	TCGCTGTCTTGCACTCTGGTGT-F	23	2OD	HPLC
	CCAATCTGCGCTTGGAGTGATAG-R	24	2OD	HPLC
NF-κB gene	TCCTGTTCGAGTCTCCATGCAG-F	23	2OD	HPLC
	GGTCTCATAGGTCCTTTTGCGC-R	23	2OD	HPLC

### 2.9 Expression analysis of p53 pathway-related genes

The mRNA expression levels of ATM, Wip1, ATR, Chk2, MDM2, p53, p21, caspase3, and NF-κB were quantified by qRT-PCR. Liver samples from mice of the same growth phase, with or without Nd(NO_3_)_3_ treatment, were homogenized, and total RNA was isolated using the aforementioned RNA extraction kit. The FastKing gDNA Dispelling RT SuperMix was used for cDNA synthesis under the following conditions: 42°C for 15 min, followed by 95°C for 3 min. Primers for qRT-PCR were designed using Primer Express Software and synthesized by Sangon Biotech (China). The qRT-PCR reaction mixture was prepared with SuperReal Premix Plus and consisted of a three-step cycling program: 95°C for 15 min, followed by 40 cycles of 95°C for 10 s, 58°C for 20 s, and 72°C for 30 s. Gene expression analysis was conducted using standard curve and quantitation methods.

### 2.10 Statistical analysis

Data were analyzed using GraphPad Prism eight and presented as means ± standard error (SE). One-way ANOVA was used to assess differences among multiple groups, followed by Tukey’s HSD for pairwise comparisons with the control group, with significance set at *p* < 0.05.

For the Benchmark Dose (BMD) analysis, we utilized the e(BMD) model with Benchmark Dose Software (BMDS, version 3.2) following USEPA guidance (Davis et al., 2011). The best-fit model was selected based on the lowest Akaike Information Criterion (AIC) to calculate parameters such as BMD, BMDL, and BMDU, representing the BMD and its 95% confidence intervals. The BMD_50_ was used for all analyses, corresponding to a 50% increase in genotoxicity test frequency over background. Data were batched by exposure route and analyzed using combined covariate BMD modeling to ensure consistency and computational feasibility. The Index and Hill model family, recommended by EFSA for continuous data analysis, were applied (Hardy et al., 2017; E.F.S.A. EFSA, 2009). Covariate analysis assumed constant model parameters for maximum response and steepness of the dose-response curve. Unique identifiers were assigned to datasets with different parameters, used as covariates for background response, efficacy, and within-group variance. For compounds with multiple datasets, the lowest BMDL value was used for MOE calculations (Elhajouji et al., 1995).

## 3 Result

### 3.1 Effect of 28-day neodymium nitrate administration on body weight of ICR mice

**FIGURE 1:**
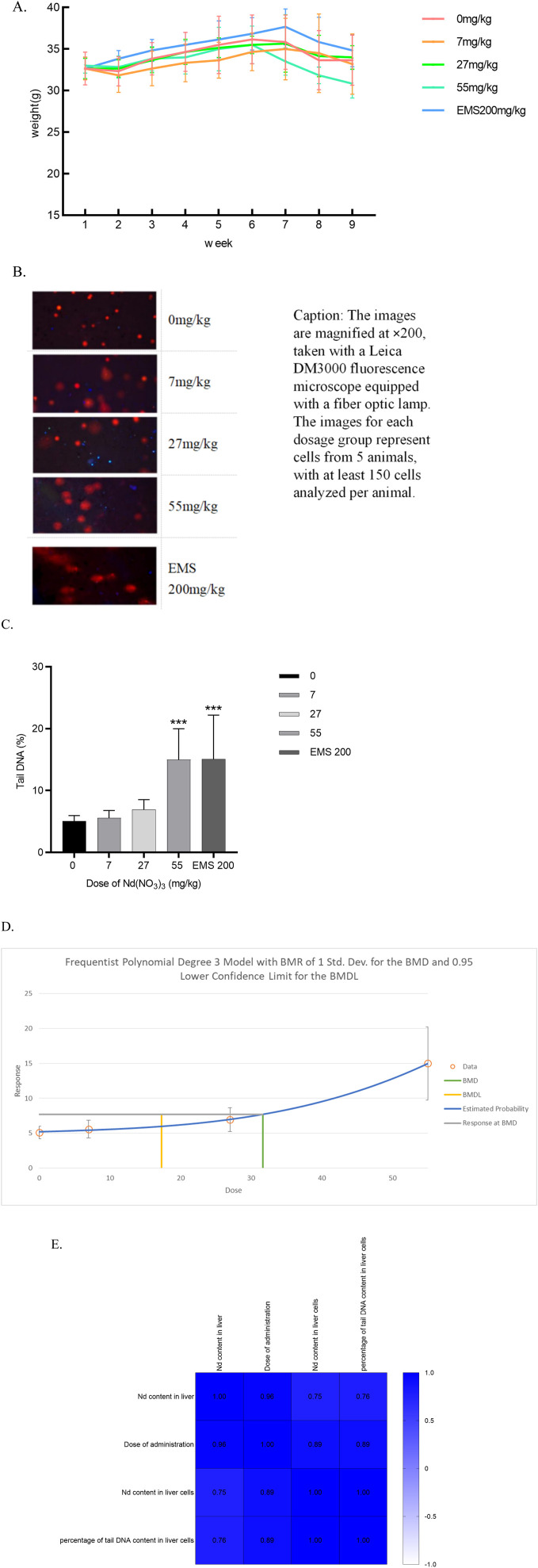
The Effects of 28-Day Neodymium Nitrate Administration on Mouse Body Weight, Liver Neodymium Content, and Genotoxicity, and Their Correlation Analysis. **(A)** The effect of 28-day neodymium nitrate administration on the body weight of mice, **(B)**. Comet assay photos of hepatocytes from ICR mice in various dosage groups, **(C)**. percentage of DNA in the tail of hepatocyte comet of male ICR mice, **(D)**. BMD of percentage of DNA in the tail of hepatocyte comet of male ICR mice, **(E)**. Correlation analysis of neodymium content in liver and liver cells after 28 days of neodymium nitrate administration, with dosing levels, and the percentage of tail DNA content in liver cells). (*: Compared with the negative control group, *p* < 0.05; **: Compared with the negative control group, *p* < 0.01; ***: Compared with the negative control group, *p* < 0.001; ****: Compared with the negative control group, *p* < 0.0001).

The weekly body weight changes of ICR mice following 28 days of continuous Nd(NO_3_)_3_ administration are depicted in Figure 1. No significant differences in body weight were observed between the treatment groups and the solvent control group (*p* > 0.05). There were no noticeable changes in the general health of the treatment groups compared to the control group. Post-administration, some individual mice exhibited signs of lethargy, reduced mobility, and dulled fur; however, these symptoms did not present a consistent pattern.

### 3.2 Determination of neodymium content in the liver of ICR mice after 28 Days of administration

#### 3.2.1 Limit of detection (LOD) and limit of quantitation (LOQ)

The method’s detection limit was established at 0.0018 μg/L based on the mean response plus three times the standard deviation of 11 blank samples’ responses. Recovery rates were between 97.2% and 111.0%, and relative standard deviations ranged from 1.63% to 7.93%.

The inductively coupled plasma mass spectrometer was calibrated using a mass spectrometry tuning solution to ensure optimal performance. A 10.0 μg/mL neodymium (Nd) standard stock solution was serially diluted with 1% nitric acid to create a calibration range of 0.05, 0.1, 0.5, and 1.0 μg/L. Indium (In) served as the internal standard. A calibration curve was generated with the *y*-axis representing the ratio of the signal values of neodymium to indium and the *x*-axis representing the neodymium content. The linear correlation coefficient was 0.9993, indicating that the method’s performance meets the detection criteria.

#### 3.2.2 Determination of neodymium content in the liver of ICR mice

After continuous administration of Nd(NO_3_)_3_ for 28 days, the changes in neodymium content in the tissues and organs of ICR mice are shown in Figure 3-18. It can be observed that: In liver, it began to significantly exceed the solvent control group from a dosage of 14 mg/kg body weight (*p* values were 0.0008, 0.0003, <0.0001, and <0.0001). In hepatocytes, the neodymium content in male mice, it began to significantly exceed the solvent control group from a dosage of 39 mg/kg body weight (*p* values were 0.0119 and <0.0001).

### 3.3 DNA damage in liver tissue cells induced by 28-day neodymium nitrate administration

In the neodymium nitrate treatment groups, DNA breaks were observed, characterized by the formation of orange-red fluorescent nuclei and comet-shaped tails in the cells. At a treatment concentration of 55 mg/kg body weight (BW), the percentage of tail DNA content in liver cells of ICR mice was significantly elevated compared to the control group, with a statistically significant difference (*p* = 0.0003). Similarly, in the positive control group treated with Ethyl Methanesulfonate (EMS) at 200 mg/kg BW, the percentage of tail DNA content in liver cells of male ICR mice was significantly higher than that of the control group (*p* = 0.0003). As shown in Figure 1. A dose-response relationship was observed as the treatment dosage of neodymium nitrate increased, with a continuous rise in the percentage of tail DNA content in mouse liver cells. The Benchmark Dose Lower Limit (BMDL) for the percentage of tail DNA content in liver cells of male ICR mice was 17.32 mg/kg, and the Benchmark Dose (BMD) value was 31.63 mg/kg. As shown in Figure 1.

### 3.4 Correlation analysis of neodymium content in liver and liver cells after 28 Days of neodymium nitrate administration

The neodymium content in liver and the dosage of administration showed significant correlations with all assessed indicators (*p* < 0.05). Notably, the percentage of DNA content in the tail of liver cells correlated significantly with both the neodymium content in liver and the liver cells (*p* < 0.05). As shown in Figure 1.

### 3.5 Effects of 28-day neodymium nitrate administration on Liver Tissue Biomarkers

#### 3.5.1 γ-H2AX content

**FIGURE 2:**
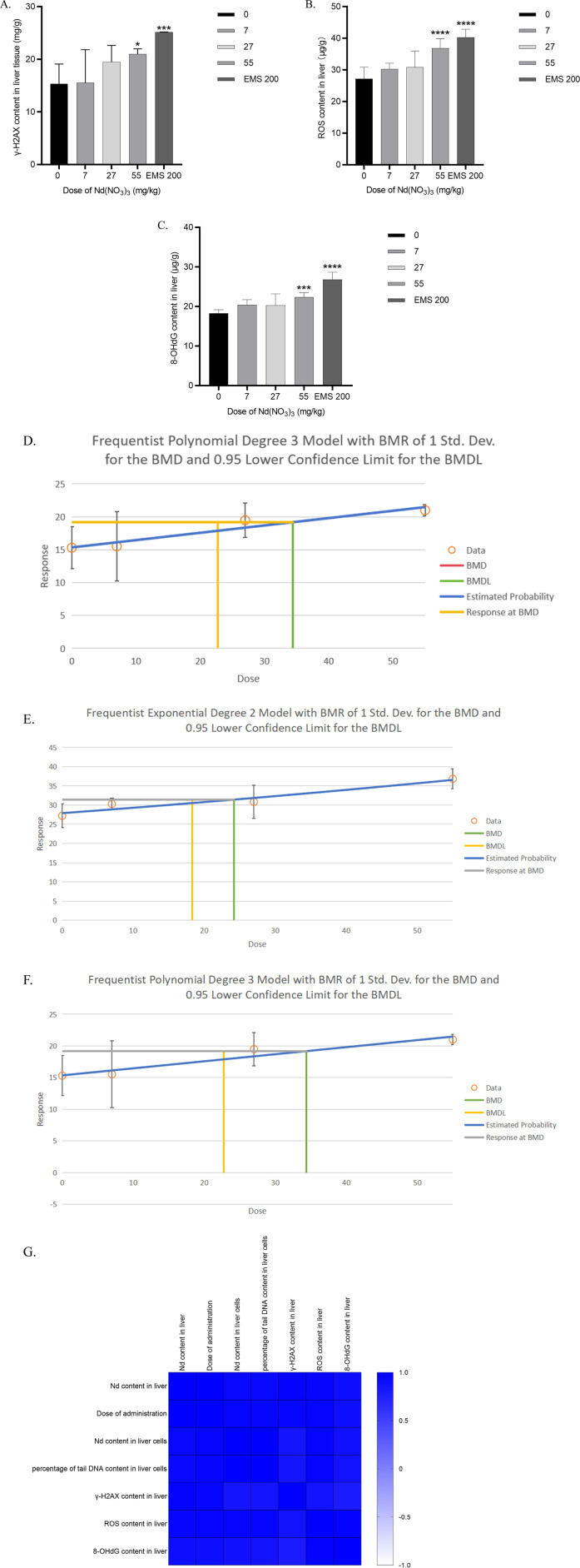
Determination of γ-H2AX, ROS and 8-OHdG content in liver tissue after 28 days of neodymium nitrate administration **(A)**. γ-H2AX content in liver tissue of male ICR mice, **(B)**. ROS content in liver tissue of male ICR mice, **(C)**. 8-OHdG content in liver tissue of male ICR mice, **(D)**. Benchmark dose (BMD) values for γ-H2AX content in liver tissue of male ICR mice, **(E)**. Benchmark dose (BMD) values for ROS content in liver tissue of male ICR mice, **(F)**. Benchmark dose (BMD) values for 8-OHdG content in liver tissue of male ICR mice, **(G)**. Correlation analysis of neodymium content in liver and liver cells, the percentage of tail DNA content in liver cells after 28 days of neodymium nitrate administration, with dosing levels, and γ-H2AX, ROS and 8-OHdG content in liver tissue).

At a dosing concentration of 55 mg/kg body weight (BW), the liver tissue γ-H2AX content in male ICR mice was significantly elevated compared to the control group (*p* = 0.0345). In the positive control group administered Ethyl Methanesulfonate (EMS) at 200 mg/kg BW, the γ-H2AX content was also significantly higher than in the control group (*p* < 0.0001). A dose-dependent increase in γ-H2AX content was observed, indicating a clear dose-response relationship. The Benchmark Dose Lower Limit (BMDL) for γ-H2AX content in liver tissue of male ICR mice was 20.74 mg/kg, with a Benchmark Dose (BMD) of 34.40 mg/kg. As shown in Figure 2.

#### 3.5.2 ROS content

At dosing concentrations of 14, 39, and 55 mg/kg BW, the liver tissue ROS content in male ICR mice was significantly higher than in the control group (*p* < 0.0001 for all doses). In the EMS positive control group at 200 mg/kg BW, ROS content was also significantly increased compared to the control group (*p* < 0.0001). A dose-response relationship was evident as the dosing concentration of neodymium nitrate increased. The BMDL for ROS content in liver tissue was 18.32 mg/kg, and the BMD was 24.21 mg/kg. As shown in Figure 2.

#### 3.5.3 8-OHdG content

At a dosing concentration of 55 mg/kg BW, the liver tissue 8-OHdG content in male ICR mice was significantly higher than in the control group (*p* < 0.0001). Similarly, in the EMS positive control group at 200 mg/kg BW, the 8-OHdG content was significantly elevated compared to the control group (*p* < 0.0001). A dose-dependent increase in 8-OHdG content was observed, consistent with a dose-response relationship. The BMDL for 8-OHdG content in liver tissue was 20.51 mg/kg, with a BMD of 29.90 mg/kg. As shown in Figure 2.

#### 3.5.4 Correlation analysis of neodymium content in liver and liver tissue biomarkers after 28 days of neodymium nitrate administration

Notably, γ-H2AX content, ROS Content and 8-OHdG Content in liver correlated significantly with the dosage of administration, the neodymium content in liver and the percentage of DNA content in the tail of liver cells (*p* < 0.05). As shown in Figure 2.

### 3.6 Effects of 28-day neodymium nitrate administration on relative RNA expression of p53 pathway genes in liver tissue

RNA extraction was performed using the Tiangen Animal Tissue and Cell Total RNA Extraction Kit (DP451) according to the manufacturer’s protocol. Reverse transcription was carried out using the Tiangen One-Step gDNA Removal cDNA Synthesis Kit (KR118). Quantitative real-time PCR (qRT-PCR) was conducted to assess mRNA expression levels using the Tiangen SuperReal PreMix Plus (SYBR Green) as the fluorescent quantitative premixed reagent, with β-actin serving as the internal control. Primers were selected based on a literature search (Zhao et al., 2011).

In male ICR mice, the relative RNA expression of the following genes showed significant increases compared to the control group at the indicated neodymium nitrate dosing concentrations:

ATM: At 27 and 55 mg/kg BW, with *p*-values of 0.0239 and 0.0004, respectively.

Wip1: At 27 and 55 mg/kg BW, with *p*-values of 0.0097 and 0.0003, respectively.

ATR: At 27 and 55 mg/kg BW, with *p*-values of 0.0030 and 0.0002, respectively.

MDM2: At 27 and 55 mg/kg BW, with *p*-values of 0.0004 and <0.0001, respectively.

p53: At 7 and 27 mg/kg BW, with a *p*-value of 0.0019 and 0.0379, respectively.

p21: At 7, 27, and 55 mg/kg BW, with *p*-values of 0.0464, 0.0070, and 0.0005, respectively.

NF-κB: At 7, 27, and 55 mg/kg BW, with *p*-values of 0.0310, 0.0020, and <0.0001, respectively.

**FIGURE 3:**
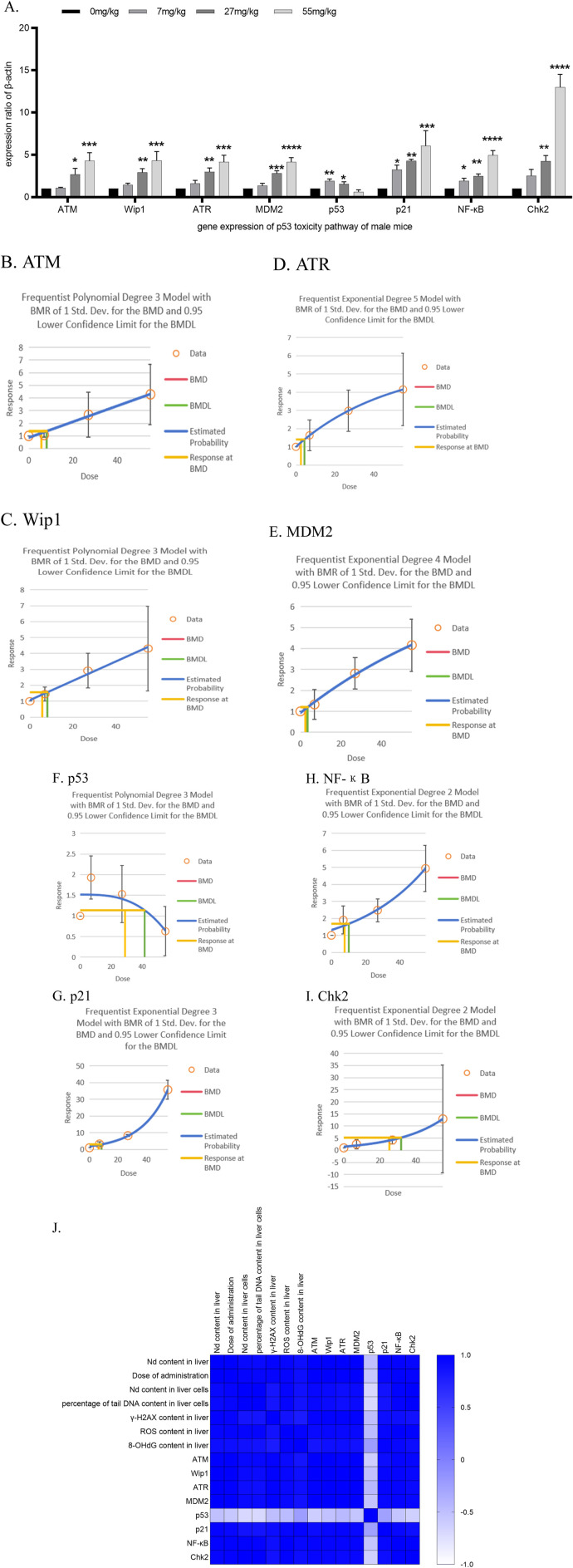
Determination of the relative RNA expression of p53 molecular pathway genes in liver tissue after 28 days of neodymium nitrate administration **(A)**. the relative RNA expression of p53 molecular pathway gene in liver tissue after 28 days of neodymium nitrate administration, **(B)**. Benchmark dose (BMD) values for ATM content in liver tissue of male ICR mice, **(C)**. Benchmark dose (BMD) values for Wip1 content in liver tissue of male ICR mice, **(D)**. Benchmark dose (BMD) values for ATR content in liver tissue of male ICR mice, **(E)**. Benchmark dose (BMD) values for MDM2 content in liver tissue of male ICR mice, **(F)**. Benchmark dose (BMD) values for p53 content in liver tissue of male ICR mice, **(G)**. Benchmark dose (BMD) values for p21 content in liver tissue of male ICR mice, **(H)**. Benchmark dose (BMD) values for NF-κB content in liver tissue of male ICR mice, **(I)**. Benchmark dose (BMD) values for Chk2 content in liver tissue of male ICR mice, **(J)**. Correlation analysis of neodymium content in liver and liver cells, the percentage of tail DNA content in liver cells, γ-H2AX, ROS and 8-OHdG content in liver tissue after 28 days of neodymium nitrate administration, with dosing levels, and the relative RNA expression of p53 molecular pathway genes in liver tissue).

Chk2: At 27 and 55 mg/kg BW, with *p*-values of 0.0065 and <0.0001, respectively. As shown in Figure 3.

All observed differences were statistically significant, indicating a dose-dependent effect of neodymium nitrate on the expression of these genes. As shown in Table 2.

**TABLE 2:** BMD Calculation for qPCR Quantitative Analysis.

p53 Molecular Pathway Gene	BMD(mg/kg)	BMDL(mg/kg)
ATM gene	8.17	5.86
Wip1 gene	5.94	3.37
ATR gene	4.40	2.61
MDM2 gene	3.63	2.39
p53 gene	51.72	29.07
p21 gene	2.84	1.04
NF-κB gene	10.22	7.76
Chk2 gene	11.32	8.72

Correlation Analysis of Neodymium Content in Liver, Liver Tissue Biomarkers and the Relative RNA Expression of p53 Molecular Pathway Genes in Liver Tissue After 28 days of Neodymium Nitrate Administration:

Notably, the relative RNA expression levels of ATM, Wip1, ATR, MDM2, p21, NF-κB, and Chk2 genes were significantly positively correlated with the dosage of administration, the neodymium content in the liver, the percentage of DNA content in the tail of liver cells, and the levels of γ-H2AX, ROS, and 8-OHdG in the liver (*p* < 0.05). In contrast, the relative RNA expression level of the p53 gene was significantly negatively correlated with the dosage of administration, the neodymium content in the liver, the percentage of DNA content in the tail of liver cells, and the levels of γ-H2AX, ROS, and 8-OHdG in the liver (*p* < 0.05). As shown in Figure 3.

## 4 Discussion

Traditional genotoxicity testing paradigms, established in the 1970s and 1980s in North America, Japan, and Europe, have been pivotal in dichotomously classifying chemicals as genotoxic or non-genotoxic (Barlow et al., 2006). This binary categorization has underpinned risk management strategies for decades. However, the advent of high-throughput testing technologies has unveiled complex, non-linear dose-response relationships, particularly at low doses (Cao et al., 2014). Aneugens like colchicine and mutagenic substances such as methyl sulfonate demonstrate curved dose-response patterns, suggesting that biological responses to these agents may entail both dose-dependent and threshold mechanisms (EFSA Scientific Committee, 2011). These insights necessitate a reevaluation of existing models and a more nuanced understanding of dose-response dynamics, especially at lower exposure levels, to accurately assess chemical safety and potential human health risks (Massarweh, 2016).

Quantitative genotoxicity risk assessment has advanced beyond qualitative methods, offering a more refined tool for evaluating health risks (Amlacher, 1988). The traditional ALARA principle has been augmented by quantitative approaches that calculate the margin of exposure (MOE), the ratio of acceptable exposure levels to actual or anticipated human exposure. This metric facilitates a more precise estimation of health risks associated with genotoxicants (Doak et al., 2007). Quantified measures like MOEs and reference doses enhance the ability of risk managers to prioritize and control genotoxic substances effectively and communicate the magnitude of risks to the public and policymakers.

In this study, 28 days of continuous neodymium nitrate (Nd(NO_3_)_3_) administration to ICR mice revealed no significant body weight changes compared to the control group, suggesting minimal toxicity within the tested dosage range (7–55 mg/kg for males). Although our ICP-MS analysis indicates the accumulation of neodymium in the liver, consistent with previous studies that highlight the liver as a primary target organ for rare earth elements, recent findings suggest that neodymium may also be excreted through bile, affecting its accumulation pattern (Wang et al., 2024). Further research is needed to clarify the distribution and long-term accumulation of neodymium in various organs.

Our findings indicate potential genotoxic effects of Nd(NO_3_)_3_ in mice, with implications for human exposure. Hepatocyte alkaline comet assays were conducted in accordance with OECD guidelines, providing precise DNA damage measurements. A quantitative genotoxicity assessment using the Benchmark Dose (BMD) approach allowed us to calculate the MOE for human health risks, offering a comprehensive evaluation of Nd(NO_3_)_3_ genotoxicity. Correlation analysis between liver neodymium concentration and genotoxicity outcomes post-Nd(NO_3_)_3_ administration revealed significant associations, suggesting a dose-dependent genotoxic effect. The biomarker analysis further confirmed Nd(NO_3_)_3_-induced genotoxicity and its DNA damaging effects on hepatocytes.

Low genotoxicity refers to the relatively low degree of damage caused to genetic material by substances or environmental factors, which may not be sufficient to immediately cause obvious biological effects, such as cell death or apparent genetic mutations. However, even low levels of genotoxicity can pose a potential risk to human health, especially when people are exposed to such substances over a long period.

Substances with low genotoxicity may cause the following types of DNA damage: 1. Single-strand breaks (SSBs): Breaks in one of the strands of the DNA double helix. 2. Base damage: Alteration of DNA bases due to chemical reactions, such as oxidation, alkylation, or deamination. 3. DNA cross-links: The formation of covalent bonds between the two complementary strands of DNA, causing DNA structure distortion. 4. DNA adducts: The binding of external chemical substances to DNA bases, altering their normal structure and function.

Reactive oxygen species (ROS) are highly reactive molecules produced during normal cellular metabolism. They play a crucial role in regulating cellular signal transduction and maintaining cellular homeostasis. However, when the balance between ROS production and elimination is disrupted, an excess of ROS can cause oxidative damage to cellular components, including lipids, proteins, and DNA. In some cases, ROS can act as inducers, inducing cellular genotoxicity such as DNA single-strand breaks (SSBs), which are a form of DNA damage. If SSBs are not repaired in time or are repaired incorrectly, they may lead to changes in genetic information. Low levels of genotoxicity, such as SSBs, trigger the DNA Damage Response (DDR) within the cell. DDR involves a series of genes and proteins that work together to recognize and repair DNA damage, or to trigger cell cycle arrest or apoptosis when necessary. Cells have dedicated sensor proteins, such as ATM (Ataxia Telangiectasia Mutated) and ATR (ATM and Rad3 related), that can recognize DNA single-strand breaks and activate downstream signaling pathways. Once the DDR pathway is activated, it triggers cell cycle checkpoints, such as p53 and Chk1/Chk2, which help to pause the cell cycle, providing time for the cell to repair DNA damage.

Changes in the expression of DDR genes play a crucial role in maintaining genomic stability. The DNA damage response (DDR) is a series of complex molecular events that cells undergo in response to DNA damage. Its purpose is to identify and repair DNA damage, or to trigger cell cycle arrest and apoptosis when the damage cannot be repaired, preventing the proliferation of damaged cells. DDR genes include a range of genes involved in the perception of DNA damage, signal transduction, and DNA repair. In situations of low genotoxicity, the expression of DDR genes may change to cope with and attempt to repair the damage.

In the context of low genotoxicity, when SSBs caused by ROS inducers are about 10%, changes in the expression of DDR genes may manifest as upregulation of genes involved in DNA repair and activation of genes involved in cell cycle control and apoptosis (such as p53, p21, etc.). These changes reflect the cell’s attempt to repair the damage and restore its normal function.

**FIGURE 4:**
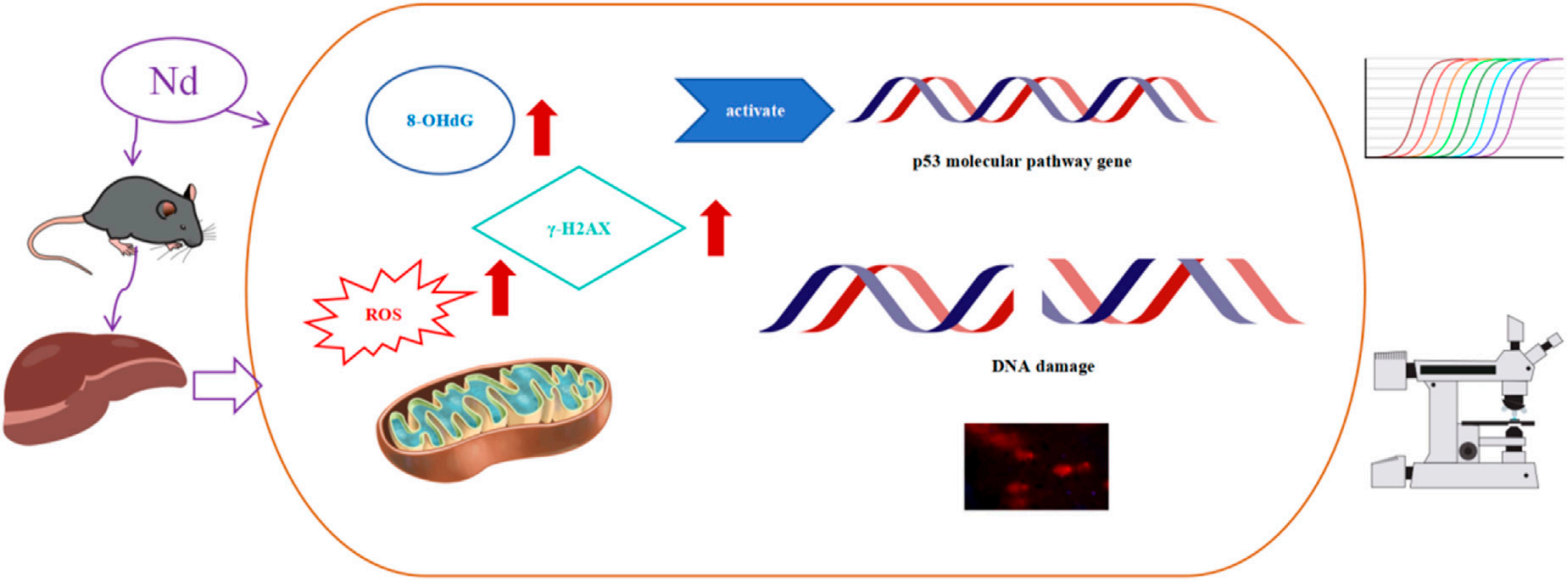
The possible mechanisms of the genotoxic effects of neodymium nitrate on mouse liver.

In this study, when there was a significant increase of 10% in ROS, the dose of Nd(NO_3_)_3_ was approximately 55 mg/kg, at which point DDR genes all exhibited significant changes. The p53 signaling pathway, a critical biomarker in genotoxicity research, was examined for its role in the molecular response to Nd(NO_3_)_3_ (Nadal, 2011; Wills et al., 2017). Our analysis of BMD (Benchmark Dose) and BMDL (Benchmark Dose Lower Limit) values for genes in the p53 pathway, including ATM, Wip1, ATR, MDM2, p53, p21, NF-κB, and Chk2, along with comet assay and genotoxicity biomarker data, identified p21, MDM2, and Wip1 as potential early indicators of DNA damage. These findings provide a theoretical foundation for understanding the genotoxicity mechanism of Nd(NO_3_)_3_, as illustrated in Figure 4.

As a cyclin-dependent kinase inhibitor, p21 is intricately linked to tumor suppression, regulating cell cycle, DNA replication, and repair processes (Zeller et al., 2016). qRT-PCR analysis of the p53 pathway and downstream genes revealed significant upregulation of ATM, ATR, Wip1, MDM2, p21, and Chk2, suggesting a close relationship between Nd(NO_3_)_3_-induced liver genotoxicity and the p53 pathway, with implications for cell cycle arrest.

Building on the previous discussions, the alkaline comet assay is introduced as a tool to confirm these mechanisms. The assay serves as a sensitive method for detecting DNA damage at the individual cell level. It is particularly adept at identifying single-strand breaks (SSBs) and, under certain conditions, double-strand breaks (DSBs). The alkaline environment of the comet assay facilitates the detection of DNA strand breaks. The DNA is more susceptible to unwinding and breakage under these conditions, which can reveal even minor damages. In the alkaline comet assay, SSBs appear as a “comet tail” with DNA fragments migrating towards the anode. DSBs, while more challenging to detect in this assay, can be inferred from a more pronounced tail or other abnormalities in the comet’s shape. DDR is triggered by DSBs as a way to maintain genomic integrity. The activation of DDR involves the phosphorylation of the histone variant H2AX at the site of the break, leading to the recruitment of repair proteins such as KU70/KU80 and DNA-PKcs in the case of non-homologous end joining (NHEJ), or RAD51 in the case of homologous recombination repair (HRR). The efficient and accurate activation of DDR is critical for cell stability. If DSBs are not repaired correctly, it can lead to chromosomal translocations, genomic instability, and potentially carcinogenesis. The DDR also plays a role in activating cell cycle checkpoints to halt cell division until the damage can be resolved. The assay can provide insights into the cell’s choice of repair pathways. For example, the persistence of a comet tail may suggest an inability to repair the damage efficiently, implicating a failure in one of the repair pathways.

It is worth noting that although SSBs induced by ROS at low genotoxicity levels may not immediately lead to severe biological consequences, long-term or cumulative low-level genotoxicity may increase the risk of cellular transformation. Therefore, understanding the mechanisms of action of ROS inducers at low genotoxicity levels and the changes in the expression of DDR genes is of great significance for the prevention and treatment of diseases related to genotoxicity.

It is noteworthy that the γ-H2AX response indicates a higher level of DNA damage compared to what is observed in the comet assay. γ-H2AX is a sensitive marker for double-strand breaks (DSBs). The formation of γ-H2AX foci occurs rapidly after DSBs are introduced into the DNA, making it an early indicator of DSBs. The presence of γ-H2AX signifies the activation of the DNA Damage Response (DDR), particularly the pathway involving the phosphorylation of H2AX by DNA-dependent protein kinase (DNA-PK), ATM, and ATR. A high level of γ-H2AX indicates a significant amount of DNA damage that the cell must address. Depending on the cell’s ability to repair the DSBs, this could lead to cell cycle arrest, senescence, or apoptosis. A significantly higher number of γ-H2AX foci would suggest more severe DNA damage. It is also important to consider other biomarkers of genotoxicity that might be elevated alongside γ-H2AX, such as the expression of other DDR genes or the presence of other types of DNA damage. The heightened response of γ-H2AX underscores the importance of using multiple assays to assess genotoxicity. Different assays detect different types or levels of DNA damage, and using them in combination can provide a more comprehensive understanding of a substance’s genotoxic potential. By acknowledging the increased sensitivity of γ-H2AX as a biomarker for DSBs and supporting this with comparative data from the comet assay and other genotoxicity assays, a stronger case can be made for the genotoxic potential of a given agent and the necessity of further investigation or precautionary measures.

The positive correlation between the relative RNA expression levels of ATM, Wip1, ATR, MDM2, p21, NF-kB, and Chk2 genes and the dosage of administration suggests that as the dosage increases, the expression levels of these genes also rise accordingly. This may imply that these genes play an important role in responding to DNA damage. The positive correlation between the expression of these genes and the neodymium content in the liver implies that the presence of neodymium may directly or indirectly affect the expression of these genes. This likely reflects the cellular stress response to neodymium-induced DNA damage. The positive correlation between the percentage of DNA content in the tail of liver cells (commonly measured in the comet assay) and gene expression further supports the link between DNA damage and changes in gene expression. γ-H2AX, ROS, and 8-OHdG are biomarkers of DNA damage. Their levels’ positive correlation with gene expression indicates that these genes may be involved in DNA damage response pathways. The negative correlation of p53 gene expression with the aforementioned parameters may suggest that under these experimental conditions, the response of p53 differs from other genes. p53 is typically involved in regulating cell cycle arrest and apoptosis, and its decreased expression may be associated with the cell’s overall response strategy to damage. These data may reveal the relative contributions of SSBs and DSBs to genotoxicity. For instance, if biomarkers associated with DSBs (such as γ-H2AX) show a stronger positive correlation with gene expression, this may indicate that DSBs play a more significant role in inducing changes in gene expression. By analyzing these correlations, it is possible to more accurately assess the extent of genotoxicity caused by SSBs and DSBs. This helps to understand the impact of different types of DNA damage on cell function and survival, as well as their potential role in disease development. In summary, these findings reveal the complexity in DNA damage responses and emphasize the potential value of different genes and biomarkers in assessing genotoxicity.

## Data Availability

The original contributions presented in the study are included in the article/Supplementary Material, further inquiries can be directed to the corresponding authors.
